# Modification of Metal-Organic Framework-Derived Nanocarbons for Enhanced Capacitive Deionization Performance: A Mini-Review

**DOI:** 10.3389/fchem.2020.575350

**Published:** 2020-11-30

**Authors:** Peng Lin, Maoxin Liao, Tao Yang, Xinran Sheng, Yue Wu, Xingtao Xu

**Affiliations:** ^1^College of Hydrology and Water Resources, Hohai University, Nanjing, China; ^2^State Key Laboratory of Hydrology-Water Resources and Hydraulic Engineering, Hohai University, Nanjing, China

**Keywords:** capacitive deionization, nanocarbon, metal-organic framework, modification, desalination

## Abstract

Capacitive deionization (CDI) is a promising electrochemical water treatment technology. Development of new electrode materials with higher performance is key to improve the desalination efficiency of CDI. Carbon nanomaterials derived from metal–organic frameworks (MOFs) have attracted wide attention for their porous nanostructures and large specific surface areas. The desalination capacity and cycling stability of MOF-derived carbons (MOFCs) have been greatly improved by means of morphology control, heteroatom doping, Faradaic material modification, etc. Despite progress has been made to improve their CDI performance, quite a lot of MOFCs are too costly to be applied in a large scale. It remains crucial to develop MOFCs with both high desalination efficiency and low cost. In this review, we summarized three modification methods of MOFCs, namely morphology control, heteroatom doping, and Faradaic material doping, and put forward some constructive advice on how to enhance the desalination performance of MOFCs effectively at a low cost. We hope that more efforts could be devoted to the industrialization of MOFCs for CDI.

## Introduction

With the increasing shortage of water resources worldwide, the exploration of new methods for water treatment has become one of the important ways to solve the problem (Xu et al., 2017b; Sun et al., 2020a,b). Capacitive deionization (CDI) is considered a promising water treatment technology with powerful competitiveness compared with reverse osmosis and electroosmosis owing to its advantages of low energy consumption, environmental friendliness, and low cost (Oren, 2008). It shows excellent performance in the fields of seawater desalination, brackish water desalination, heavy metal ion removal (Hou et al., 2018), and element enrichment. So far, numerous materials (especially carbon materials) have been developed for CDI electrodes, including activated carbon (Wang et al., 2013; Luo et al., 2019), activated carbon nanofiber (ACF) (Wang et al., 2012), carbon aerogel (CA) (Jung et al., 2007), carbon nanotubes (CNT) (Wang et al., 2011), graphene (Xu et al., 2016b; Huang et al., 2019), ordered mesoporous carbons (OMCs) (Duan et al., 2015; Xu et al., 2019c), etc. Among them, graphene is undoubtedly the most promisingly studied electrode material for CDI mainly owing to its large specific area, low cost, and abundance (Li et al., 2012). However, its poor salt adsorption capacity (SAC) limits its further application. The development of CDI needs, first and foremost, low-cost and high-efficiency electrodes (AlMarzooqi et al., 2014).

Carbon nanomaterials derived from metal–organic frameworks (MOFs) have attracted wide attention recently (Chaikittisilp et al., 2013; Xu et al., 2017a). Thanks to the porous structures and tailored compositions of precursors (Yaghi and Li, 1995; Wang, Z., et al., 2019), MOF-derived carbons (MOFCs) show adjustable pore structures, large specific surface areas, and good conductivity, giving them unparalleled CDI performance. Since Yang et al. demonstrated that carbon derived from IRMOF-1 has the potential as a high-performance CDI electrode material (Yang et al., 2014), more and more MOFs have been used for producing CDI electrodes, including the well-known zeolitic imidazolate frameworks (ZIFs) (Liu et al., 2015b; Wang et al., 2017; Gao et al., 2018), Materials Institute Lavoisier (MILs) (Xu et al., 2016a; Wang, K., et al., 2019), and MOF-5 (Chang et al., 2015). Modifications, such as morphology control, heteroatom doping (Wang et al., 2014; Xu et al., 2015), and Faradaic material doping, have been further studied to construct nanomaterials with more reasonable structures and compositions. As a result, the SAC and cycling stability of MOFCs have been greatly improved. Nevertheless, a considerable portion of MOFCs are costly due to their complex synthesis and expensive precursors, which limits their application in a large scale. The efficient and low-cost modification of MOFCs still needs to be systematically explored.

**Figure 1:**
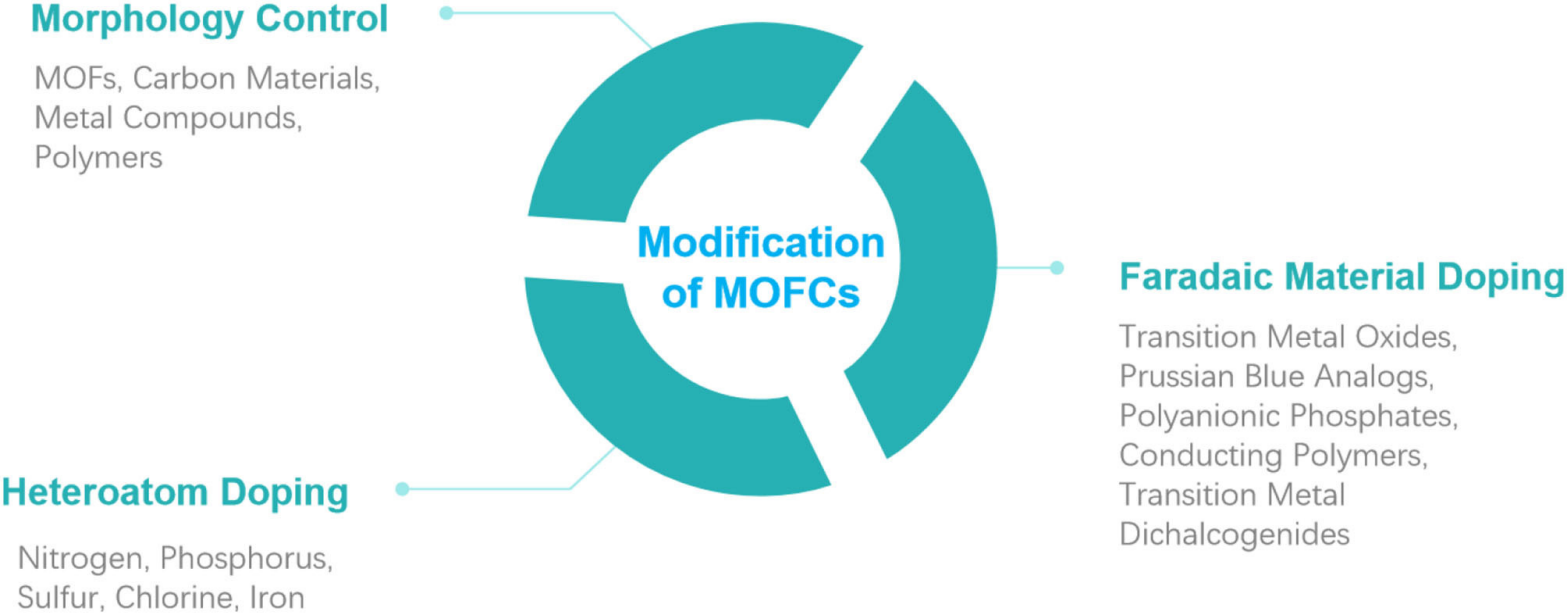
Overview of modification methods of metal–organic framework (MOF)-derived carbons.

In this paper, the principle of CDI is given, including its adsorption mechanism and requirements for electrode materials. Thereafter, three common modification methods in the aspects of morphology control by template, element doping, and Faradaic material doping are summarized (Figure 1). Moreover, we put forward some advice on cost control and discuss the future development direction of MOFCs for the desalination industry.

## The Principle of CDI

A typical CDI cell consists of two electrodes placed in parallel and saline water between them. The electrodes adsorb ions from saline water when charged and release ions when discharged, so as to desalinate feed water or recycle electrodes. The electrodes can be categorized into non-Faradaic electrodes and Faradaic electrodes according to the ion adsorption mechanism (Chen et al., 2020; Lu et al., 2020). In most carbon-based CDI processes, ions are usually stored in the electric double layers (EDLs) formed within the pores of porous electrodes without the occurrence of Faradaic reactions. For efficient and rapid desalination, electrode materials therefore should meet at least the following properties: (1) large specific surface area for ion storage and suitable pore structure for rapid migration of ions, (2) high conductivity for rapid transfer of electrons within the electrodes, (3) stable electrochemical property for cycling stability, and (4) good hydrophilicity (Yin et al., 2013; Liu et al., 2015a, 2017; Tang et al., 2019). To achieve these aims, morphology control and heteroatom doping have been frequently used. Aside from the commonly used non-Faradaic electrodes, Faradaic electrodes are also utilized to store ions mainly based on Faradaic reaction, which have attracted wide attention for their typical high SAC and cycling stability (Ding, Z., et al., 2019).

## Modification of MOFCS for Enhanced Performance

### Morphology Control With Templates

Although MOFCs have high specific surface areas and high porosities, most MOF crystals are dissociative and solid particles, which can lead to poor electrical conductivity and low accessible surface area (Tang et al., 2016; Xu et al., 2020a). Morphology control with templates, including MOF templates and external templates (e.g., carbon materials, metal compounds, polymers), may be an effective method to optimize the nanostructures and composition of MOFCs (Dang et al., 2017; Xu et al., 2019b). ZIF-8 is a typical subfamily of MOFs that has been widely investigated for CDI application. Liu et al. prepared porous carbon polyhedrons (PCPs) through direct carbonization of ZIF-8, which showed an improved desalination performance (with a SAC of 13.86 mg g^−1^) and stability compared with commercial AC (Liu et al., 2015b). Subsequently, Xu et al. reported hierarchical porous carbon nanotubes (CNTs)/PCP hybrid (hCNTs/PCP) fabricated via *in situ* insertion of CNTs in ZIF-8 with a subsequent pyrolysis process. Thanks to its novel CNT-inserted-PCP porous structure, high specific surface area, and good electrical conductivity, the resultant hCNTs/PCP exhibited a high SAC of 20.5 mg g^−1^ and stable cycling stability (Xu et al., 2016d). After that, Xu et al. synthesized integrated MOF tubes by controlled growth of ZIF-8 nanocrystals on 3D polymeric fibers with the subsequent dissolution of template (Supplementary Figure 1). Afterwards, self-standing nitrogen-doped carbon tubes (NCTs) with an ultrahigh SAC of 56.9 mg g^−1^ were obtained by thermal conversion of the resulting MOF tubes (Xu et al., 2020b). The external templates can tune the morphology of MOFCs effectively; however, their market price might not be acceptable for practical application and sometimes require complicated template removal operation (Dang et al., 2017). More versatile and cheaper templates that effectively controlled the morphology are needed (Dutta et al., 2016; Xu et al., 2016c).

### Heteroatom Doping

Heteroatom doping is a common modification method for improving the electrochemical performance of carbon materials (Li et al., 2018). Non-metallic elements or metal ions can be evenly doped in MOFCs by simple carbonization of MOF precursors containing target elements, which would contribute to enhancing the comprehensive properties of carbon materials including conductivity, hydrophilicity, and stability (Kurak and Anderson, 2009; Zheng et al., 2011; Cheng et al., 2019; Xu et al., 2019a). Gao et al. synthesized nitrogen-doped graphitic carbon polyhedrons (NGCPs) by direct carbonization of ZIF-8. NGCPs show a maximum SAC of 17.73 mg g^−1^ and high salt adsorption rate of 4.14 mg g^−1^ min^−1^ and good regeneration performance (Gao et al., 2018). Zhang et al. prepared N, P, S co-doped hollow carbon polyhedron (denoted as ZIF-8@PZS-C) derived from ZIF-8-based core–shell nanocomposites (denoted as ZIF-8@PZS). The resultant ZIF-8@PZS-C displayed an improved electrical conductivity, excellent hydrophilic, and high SAC of 22.19 mg g^−1^ (Zhang et al., 2018). Considering the performance fading of conventional carbon materials caused by the formation of H_2_O_2_ due to the reduction of dissolved oxygen in nature saline water, the introduction of oxygen reduction mechanism will effectively improve the stability of MOFCs (Luo et al., 2019). Xu et al. prepared nitrogen–iron-doped carbon tubes (3D-FeNC tubes) derived from the 3D interconnected MOF tubes (Supplementary Figure 2). Thanks to its well-defined structure and enhanced oxygen reduction ability, the 3D-FeNC tubes achieved both excellent salt removal ability and cycling performance in oxygenated saline water (Xu et al., 2020a). The research reveals that high-performance oxygen reduction catalysts, such as Fe, N, and other heteroatom-doped carbon materials (Zhang et al., 2020), can significantly improve the continuous desalination performance of CDI. Heteroatom doping enables MOFCs with higher desalination capacity, faster adsorption rate, and more importantly, better stability. Dissolved oxygen ubiquitous in natural water will eventually cause the performance fading of carbon materials. By simply doping, the stability of MOFCs can be greatly improved, which contributes to their practical application for the desalination industry.

### Faradaic Material Doping

Even though great progress has been made in improving the CDI performance of MOFCs based on EDLs, further improvement of SAC seems hard to achieve due to the limitation of physical charge adsorption capacity (Suss et al., 2015; Zhao et al., 2019). Inspired by the booming field of energy storage such as sodium-ion battery and supercapacitor (Liu et al., 2020). Faradaic materials have been investigated for CDI and proved to be promising candidates with high SAC and cycling stability (Tang, W., et al., 2019). Widely studied Faradaic materials include transition metal oxides (e.g., MnO_2_, TiO_2_, Na_4_Ti_9_O_20_), Prussian blue analogs, polyanionic phosphates [e.g., FePO_4_, NaTi_2_(PO_4_)_3_, Na_3_V_2_(PO_4_)_3_], conducting polymers (e.g., polypyrrole, polyaniline), MXenes, transition metal dichalcogenides, and so on (Qin et al., 2019, 2020; Yu et al., 2019). Yang et al. prepared hierarchically porous carbon-coated zirconium oxide nanocubes (HCZ) derived from metal–organic framework (Zr-UiO-66) for CDI electrodes. The asymmetrical cell composed of HCZ negative electrode and AC positive electrode showed a remarkable SAC of 55.17 mg g^−1^ in 250 mg L^−1^ aqueous sodium chloride solution at 1.4 V (Yang and Luo, 2019). Ding et al. reported a titanium dioxide/porous carbon composite (TiO_2_@PC) derived from MIL-125 (Ti) for a membrane CDI. A synergy of high pseudocapacitance and good oxidation resistance endows the anatase TiO_2_@PC (annealed at 600°C) with an improved SAC of 46.7 mg g^−1^ at 10 mA g^−1^ and stable cycling performance over 50 cycles (Ding, M., et al., 2019). Wang et al. prepared MIL-125 (Ti)-derived NaTi_2_(PO_4_)_3_/carbon (NTP/C) composite as electrode materials for hybrid CDI (HCDI; Supplementary Figure 3). Due to the unique porous structure, high specific surface area, and good electrical conductivity of NTP/C, the HCDI system with NTP/C composite cathode and AC anode exhibited an excellent desalination performance with a high SAC of 167.4 mg g^−1^ and good desalination ability (Wang, K., et al., 2019). The experimental results reveal that it is an effective strategy to prepare Faradaic electrodes with good conductivity and high CDI performance derived from MOFs. To develop efficient, cheap, and safe Faradaic MOFC-based electrodes, more synthetic strategies of carbon materials combining MOFs with Faradaic materials need to be investigated.

As we discussed above, most MOFCs with high CDI performance usually involve controlled morphology, heteroatom doping, and Faradaic material doping. These modification methods are applied comprehensively in the synthesis of MOFCs with the purpose to optimize the nanostructure and composition of carbon materials, so as to achieve faster adsorption rate, higher SAC, and better cycling stability. The cases mentioned above with synthesis procedures and CDI performances are listed in Table 1.

**Table 1:** Typical cases of carbon electrodes derived from MOFs.

**Electrode**	**Precursor**	**Template**	**Heteroatom**	**Faradaic Material**	**Processing**	**Electrochemical Properties**	**Desalination Performance**	**Cycling Stability**
IRMOF-1-derived Carbon (Yang et al., 2014)	IRMOF-1	/	/	/	Solvent evaporation method; 900°C, nitrogen	~138 F g^−1^, 2 mV s^−1^, 1 M NaCl	~11 mg g^−1^, 1.2 V, 585 mg L^−1^	Not available
PCPs (Liu et al., 2015b)	ZIF-8	/	N	/	Chemical reaction at room temperature; 1,200°C, nitrogen, acid etching	275.69 F g^−1^, 1 mV s^−1^, 1 M NaCl	13.86 mg g^−1^, 1.2 V, 500 mg L^−1^	No obvious electrosorption capacity declination after 30 cycles
Carbon Polyhedron and carbon Nanotube Hybrids (Gao et al., 2018)	ZIF-67/carbon nanotubes	ZIF-67	N	Co_x_O_y_	Chemical reaction at 40°C; CVD treatment	343 F g^−1^, 10 mV s^−1^, 6 M KOH	7.08 mg g^−1^, 1.2 V, 500 mg L^−1^	Not available
Shuttle-like porous carbon rods (Xu et al., 2016a)	MIL-88 (Fe)	/	/	/	Hydrothermal method; 900°C, nitrogen; acid etching	223.2 F g^−1^, 1 M NaCl	16.2 mg g^−1^, 1.2 V, 1,000 mg L^−1^	95.1% after 30 cycles
NTP/C (Wang, K., et al., 2019)	MIL-125 (Ti)/NaH_2_PO_4_	MIL-125 (Ti) derived TiO_2_/carbon	/	NaTi_2_(PO_4_)_3_	Solvothermal method; 600°C, nitrogen; solvothermal, 700°C, nitrogen	164.8 F g^−1^, 10 mV s^−1^, 1 M Na2SO4	167.4 mg g^−1^, 1.8 V, 3,000 mg L^−1^	90% after 30 cycles
Porous carbon (Chang et al., 2015)	MOF-5	/	/	/	Chemical reaction at 85°C; 900°C, vacuum	107.74 F g^−1^, 50 mV s^−1^, 0.5 M NaCl	9.39 mg g^−1^, 1.2 V, 500 mg L^−1^	97.5% after 10 cycles
3D-FeNC tubes (Xu et al., 2020a)	PAN@ZIF' fiber	Zn/PAN fibers	N, Fe	/	Electrospinning method, LBL growth method, template dissolution; 900°C, nitrogen	E_onset_: 0.98 V, E_1/2_: 0.877 V, 10 mV s^−1^, 0.1 M KOH	40.7mg g^−1^, 1.2 V, 3,500 mg L^−1^	93.82% after 200 cycles (oxygenated water)
hCNTs/PCP (Xu et al., 2016b)	CNTs/ZIF-8	CNTs	N	/	*in situ* insertion of CNTs in ZIF-8; 1,000°C, nitrogen	104.2 F g^−1^, 5 mV s^−1^, 1 M NaCl	20.5 mg g^−1^, 1.2 V, 1,000 mg L^−1^	No obvious electrosorption capacity declination after 30 cycles
NCTs (Xu et al., 2020b)	PAN@ZIF-8	PAN/Zn(Ac)_2_	N	/	Electrospinning, LBL growth method, template dissolution; 900°C, nitrogen	~292 F g^−1^, 10 mV s^−1^, 1 M NaCl	56.9 mg g^−1^, 1.2 V, 3,500 mg L^−1^	96.9% after 50 cycles
NGCPs (Gao et al., 2019)	ZIF-8	/	N	/	Chemical reaction at room temperature; 1,000°C, nitrogen (low pressure)	307.4 F g^−1^, 10 mV s^−1^, 1 M NaCl	17.73 mg g^−1^, 1.4 V, 500 mg L^−1^	90.8% after 10 cycles
ZIF-8@PZS-C (Zhang et al., 2018)	ZIF-8@PZS	ZIF-8	N, P, S	/	Electrostatic interaction PZS coating; 900°C, nitrogen, acid etching	333 F g^−1^, 1 mV s^−1^, 0.5 M NaCl	22.19 mg g^−1^, 1.2 V, 500 mg L^−1^	99% after 20 cycles
HCZ (Yang and Luo, 2019)	UiO-66	/	/	ZrO_2_	Hydrothermal method; 900°C, nitrogen	128 F g^−1^, 5 mV s^−1^, 1 M NaCl	55.17 mg g^−1^, 1.4 V, 250 mg L^−1^	95.3% after 6 cycles
TiO_2_@PC (Ding, M., et al., 2019)	MIL-125 (Ti)	/	/	TiO_2_	Chemical reaction at room temperature; 600°C, argon	~260 F g^−1^, 10 mV s^−1^, 1 M NaCl	46.7 mg g^−1^, 10 mA g^−1^, 1,000 mg L^−1^	No obvious electrosorption capacity declination after 54 cycles

## Conclusions and Outlook

As a potential water treatment technology, CDI is progressively making its path to the desalination industry. In this process, the first and most important is the development of high-efficiency and low-cost electrode materials. Nanocarbon materials derived from metal–organic frameworks have become one of the most promising candidates for their highly designable precursors. Thanks to the application of creative modification methods, breakthroughs have been made in the CDI performance of MOFCs.

Nevertheless, promotion of desalination efficiency is merely the first step of industrialization, the next will be the control of cost. Generally, the synthesis of MOFCs should select a wide range of cheap raw materials and simple synthetic routes. For example, MILs composed of metal ions such as iron, titanium, manganese, and organic ligands such as fumaric acid and terephthalic acid may be an ideal choice due to their low cost, safety, and high specific surface area. In terms of morphology control, other than the template strategies mentioned above, more methods need to be investigated. Nitrogen doping is a common modification method of MOFCs with a main consideration of nitrogen source. In addition to nitrogen-containing MOFs, cheap external nitrogen sources such as urea and ammonia are also worth considering. In the aspect of MOFC-based Faradaic electrode, transition metal oxides (Kai et al., 2017) and polyanionic phosphates with low price and high salt adsorption ability and are environmentally friendly hold great potential.

In summary, MOFCs are one of the most promising electrode materials for CDI. The further developing target is to achieve higher SAC, faster desalination rate, higher cycling stability, environmental friendliness, and lower cost. Considering that recent studies have revealed the outstanding performance of hybrid CDI with Faradaic negative electrodes, Faradaic material doping might become a mainstream modification method. Moreover, since the current CDI positive electrode materials are still carbon materials, it is vital to improve the non-Faraday desalination performance of MOFCs through morphology control and element doping. It can be expected that the combination of Faradaic mechanism and non-Faradaic mechanism by selecting appropriate modification methods of MOFCs would give CDI better desalination performance.

## Author Contributions

PL, ML, XS, and YW: proposal and writing. TY and XX: revising and guidance. All authors contributed to the article and approved the submitted version.

## Conflict of Interest

The authors declare that the research was conducted in the absence of any commercial or financial relationships that could be construed as a potential conflict of interest.
