# Supplementary material for: Modification of Metal-Organic Framework-Derived Nanocarbons for Enhanced Capacitive Deionization Performance: A Mini-Review
Source: Front Chem. 2020 Nov 30;8:575350. doi: 10.3389/fchem.2020.575350 (PMC7734083; doi:10.3389/fchem.2020.575350)
Supplement: Supplementary file 1 [file Table_2.DOCX]

Supplementary Material for

Modification of Metal-organic Frameworks derived Carbon for EnhancedDesalination Performance: A Mini Review

Peng Lin^1^, Maoxin Liao,^1^ Tao Yang^1,2*^ Xinran Sheng,^1^ Yue Wu,^1^ Xingtao Xu^1*^

^1^College of Hydrology and Water Resources, Hohai University, Nanjing, China

^2^State Key Laboratory of Hydrology-Water Resources and Hydraulic Engineering, Hohai University, 1 N. Xikang Rd., Nanjing 210-098

*** Correspondence:**Corresponding Author
[tao.yang@hhu.edu.cn](mailto:tao.yang@hhu.edu.cn); [xingtao.xu@hhu.edu.cn](mailto:xingtao.xu@hhu.edu.cn)


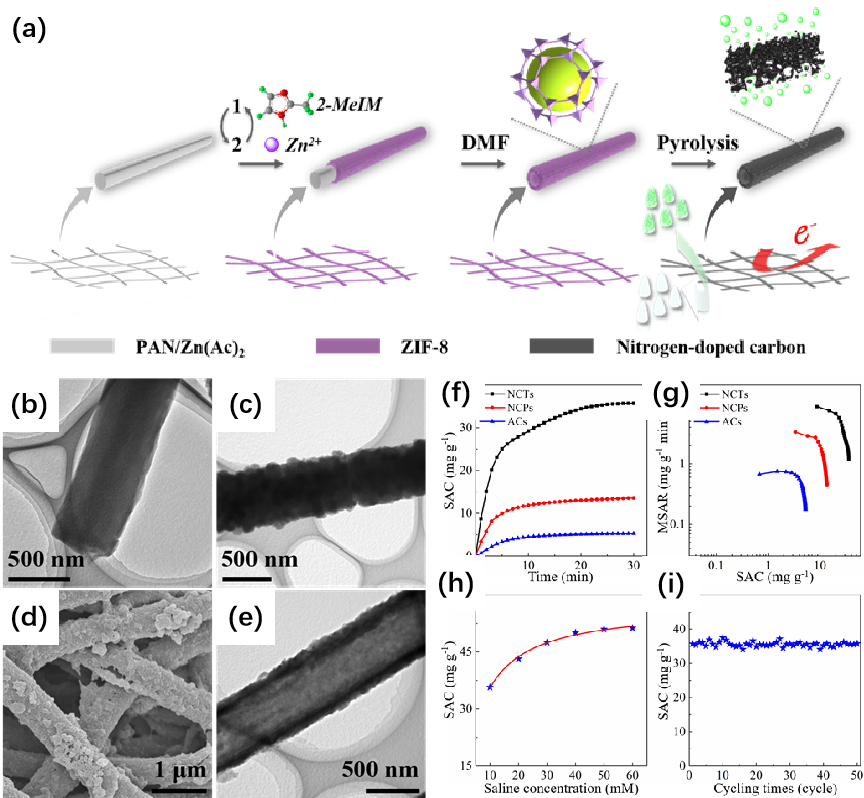


**Figure S1.** (a) Schematic diagram for the synthetic procedure of MOF-derived NCTs; (b), (c) TEM images of (b) PAN/Zn(Ac)_2_ composite fiber and (c) PAN@ZIF-8 fibers; (d) FESEM and (e) TEM images of ZIF-8 tubes; (f) SAC variations and (g) CDI Ragone plots of NCTs, NCPs and ACs; (h) SAC value vs. saline concentration and (i) cycling desalination performance of NCTs. (Reproduced with permission from (Xu et al., 2020b))


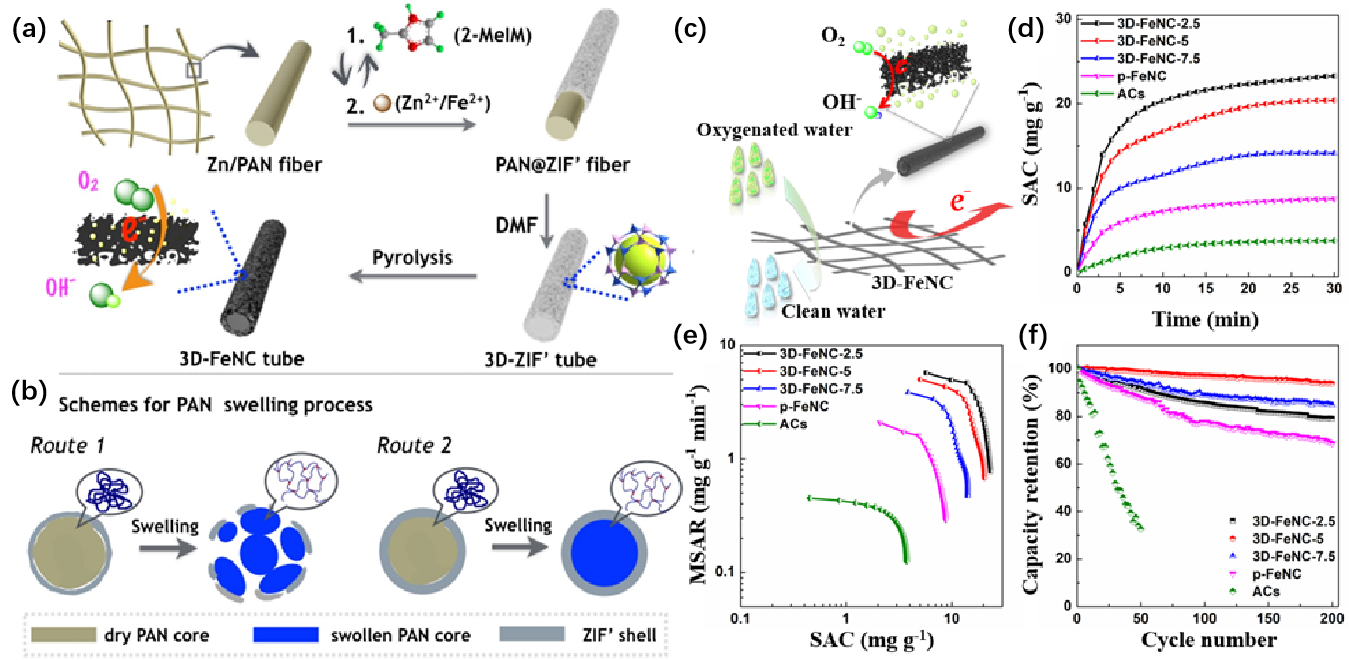


**Figure S2.** (a) Schematic illustrations showing the syntheses of 3D-ZIF’ and 3D-FeNC; (b) Schematic illustrations showing the syntheses of 3D-ZIF’ and 3D-FeNC. (c) Cross-sectional illustration depicting the dissolution of Zn/PAN core in hot DMF solvent; (d) Schematic representation of CDI process in oxygenated saline water using 3D-FeNC tubes; (e) SAC variations; (f) CDI Ragone plots, and (f) CDI cycling performances of 3D-FeNC-y (y=2.5, 5 and 7.5), pFeNC, and ACs in oxygenated saline water (5 mM). (Reproduced with permission from (Xu et al., 2020a))


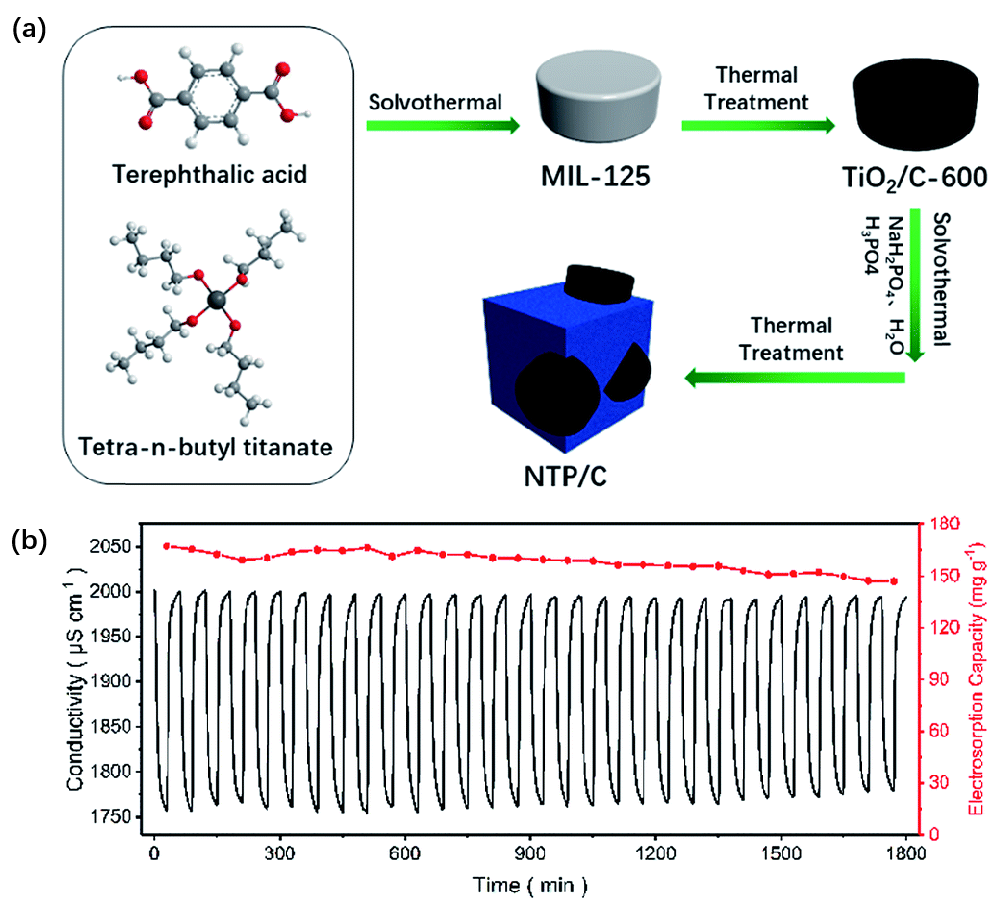


**Figure S3.** (a) Schematic illustration of the synthesis process of NTP/C composite; (b) Long-term cycling desalination experiment of HCDI. (Reproduced with permission from (Wang et al., 2019))

**References**

Wang, K., Liu, Y., Ding, Z., Li, Y., Lu, T., and Pan, L. (2019). Metal–organic-frameworks-derived NaTi 2 (PO 4) 3/carbon composites for efficient hybrid capacitive deionization. *Journal of materials chemistry A* 7(19)**,** 12126-12133.

Xu, X., Tang, J., Kaneti, Y.V., Tan, H., Chen, T., Pan, L., et al. (2020a). Unprecedented capacitive deionization performance of interconnected iron–nitrogen-doped carbon tubes in oxygenated saline water. *Materials Horizons*.

Xu, X., Yang, T., Zhang, Q., Xia, W., Ding, Z., Eid, K., et al. (2020b). Ultrahigh capacitive deionization performance by 3D interconnected MOF-derived nitrogen-doped carbon tubes. *Chemical Engineering Journal* 390**,** 124493.
